# Validation of Two Automatic Blood Pressure Monitors With the Ability to Transfer Data via Bluetooth

**DOI:** 10.2196/12772

**Published:** 2019-04-17

**Authors:** Madeleine Wetterholm, Stephanie Erika Bonn, Christina Alexandrou, Marie Löf, Ylva Trolle Lagerros

**Affiliations:** 1 Clinical Epidemiology Unit Department of Medicine Solna Karolinska Institutet Stockholm Sweden; 2 Department of Biosciences and Nutrition Karolinska Institutet Stockholm Sweden; 3 Obesity Center Academic Specialist Center Stockholm Health Services Stockholm Sweden

**Keywords:** blood pressure monitors, diabetes mellitus, type 2, hypertension, methods, mHealth, self-care, self-management

## Abstract

**Background:**

Patients with chronic diseases are in need of regular health controls. Diabetes mellitus type 2 is currently the most prevalent chronic metabolic disease. A majority of diabetic patients have at least one comorbid chronic disease, where hypertension is the most common. The standard for blood pressure (BP) measurement is manual BP monitoring at health care clinics. Nevertheless, several advantages of self-measured BP have been documented. With BP data transfer from an automatic BP monitor via Bluetooth to software, for example, a smartphone app, home measurement could effectively be integrated into regular care.

**Objective:**

The aim of this study was to validate two commercially available automatic BP monitors with the ability to transfer BP data via Bluetooth (Beurer BM 85 and Andersson Lifesense BDR 2.0), against manual BP monitoring in patients with type 2 diabetes.

**Methods:**

A total of 181 participants with type 2 diabetes were recruited from 6 primary care centers in Stockholm, Sweden. BP was first measured using a manual BP monitor and then measured using the two automatic BP monitors. The mean differences between the automatic and manual measurements were calculated by subtracting the manual BP monitor measurement from the automatic monitor measurement. Validity of the two automatic BP monitors was further assessed using Spearman rank correlation coefficients and the Bland-Altman method.

**Results:**

In total, 180 participants, 119 men and 61 women, were included. The mean age was 60.1 (SD 11.4) years and the mean body mass index was 30.4 (SD 5.4) kg/m^2^. The mean difference between the Beurer BM 85 and the manual BP monitor was 11.1 (SD 11.2) mmHg for systolic blood pressure (SBP) and 8.0 (SD 8.1) mmHg for diastolic blood pressure (DBP). The mean difference between the Andersson Lifesense BDR 2.0 and the manual BP monitor was 3.2 (SD 10.8) mmHg for SBP and 4.2 (SD 7.2) mmHg for DBP. The automatic BP measurements were significantly correlated (*P*<.001) with the manual BP measurement values (Andersson Lifesense BDR 2.0: *r*=0.78 for SBP and *r*=0.71 for DBP; Beurer BM 85: *r*=0.78 for SBP and *r*=0.69 for DBP).

**Conclusions:**

The two automatic BP monitors validated measure sufficiently accurate on a group level, with the Andersson Lifesense BDR 2.0 more often falling within the ranges for what is acceptable in clinical practice compared with the Beurer BM 85.

## Introduction

In recent years, there has been a rapid development in information and communication technology including the availability of digital devices for self-measurements and health promoting apps. Today, more and more patients are asking for digital solutions. Despite this, apps and devices for home measurement are seldom integrated into regular health care. Given the importance of self-care in the therapy of most chronic diseases, tools for self-measurement have the potential to play a greater role than they do today. Furthermore, the use of new technology such as mobile health, defined by the World Health Organization as medical or public health practice that is supported by mobile devices [1], could be a way to optimize care as a large number of patients can be reached at a lower cost.

Diabetes mellitus type 2 is currently the most prevalent chronic metabolic disease, and the number of cases is increasing worldwide. In 2015, the global prevalence of diabetes mellitus in the adult population (20-79 years) was 8.8%, and it is expected to rise to 10.4% by 2040 [2]. A majority of diabetic patients have at least one comorbid chronic disease of which hypertension, a powerful predictor of cardiovascular risk, is the most common [3,4].

Today, the standard method for diagnosis of hypertension as well as for blood pressure (BP) control in antihypertensive-treated patients is manual BP monitoring at health care clinics. Nevertheless, several advantages of self-measured BP have been documented [5]*.* For example, measurement at home can provide a more realistic appraisal of habitual BP than that can be obtained at a health care clinic, that is, eliminate the risk of the so-called *white coat hypertension* when the BP is high only in the clinical setting [6]*.* In addition, studies have also shown that the opposite, masked hypertension, that is, normal BP when measured at a clinic, but high BP when measured at home, is associated with an increased cardiovascular risk similar to the risk of patients with persistent hypertension [7,8]. As patients with diabetes have a high prevalence (47%) of masked hypertension [9], the need for validated automatic BP monitors in this specific population is of great importance.

Self-measurement at home could possibly improve patient adherence to both BP controls and treatment [10,11]. However, reporting of self-measured BP can be modified by the patients if the values for some reason do not seem suitable to them [12]. With automatic data transfer via Bluetooth to software, for example, a smartphone app, reporting bias as well as misreporting can be avoided. However, commercial automatic BP monitors are seldom validated, and to the best of our knowledge, no automatic BP monitor with data transfer via Bluetooth has been validated in patients with type 2 diabetes previously. Thus, in this study, we set out to validate two on the Swedish market commercially available automatic BP monitors (Beurer BM 85 Bluetooth and Andersson Lifesense BDR 2.0), with the ability to transfer data via Bluetooth, against manual BP monitoring in patients with type 2 diabetes.

## Methods

### Recruitment of Participants

This study was performed using BP data collected at baseline from all participants in the DiaCert-study, a randomized controlled trial of patients with type 2 diabetes. The study design has been described in detail previously [13]. A total of 181 participants were recruited from 6 primary care centers in Stockholm, Sweden. Inclusion criteria were as follows: being diagnosed with diabetes type 2, age above 18 years, being able to read and understand Swedish, being able to walk, and having access to and being able to use a smartphone. Overall, one participant did not have data on BP. Due to battery discharge or arm circumference larger than the recommended for the BP monitor cuffs, that is, more than 36 cm for Beurer BM 85 and more than 32 cm for Andersson Lifesense BDR 2.0, 11 participants did not have data from Beurer BM 85 and 25 participants did not have data from Andersson Lifesense BDR 2.0. In total, BP was measured using Beurer BM 85 in 169 participants and using Andersson Lifesense BDR 2.0 in 155 participants. All participants provided written consent before participating in the study. The study was approved by the Regional Ethical Review Board, Stockholm, Sweden (Dnr: 2016/2041-31/2; 2016/99-32; 2017/1406-32; 2018/286-32).

### The Procedure

BP, weight, height, and waist circumference were measured by study personnel at the baseline meeting. This has been described in detail previously [13]. Smoking status (never, former, or current) was assessed through a questionnaire. The BP measurements were performed after at least 5 min of rest. Participants were seated with their legs uncrossed in a quiet room, and they were instructed to avoid talking during the procedure. The upper left arm of each participant was used for the BP measurement. BP was first measured once using the manual BP monitor and then measured once using both automatic BP monitors with no specific order.

### The Automatic Monitors

The monitors Beurer BM 85 Bluetooth (Beurer GmbH. Ulm, Germany) and Andersson Lifesense BDR 2.0 (Guangdong Transtek Medical Electronics Co. Ltd. Zhongshan, China) are automatic devices for measuring BP at the upper arm. Both monitors can transfer data via Bluetooth to digital tools.

Beurer BM 85 has a pressure range of 0 to 300 mmHg and a memory capacity of 60 measurements for 2 users. It can calculate the average value of all saved measures as well as the average of morning and evening measurements during the last 7 days. Systolic blood pressure (SBP) and diastolic blood pressure (DBP) as well as the heart rate are displayed on a liquid crystal digital (LCD) display. The monitor can identify an irregular heartbeat, which is then displayed with a symbol on the LCD screen. The included standard cuff for Beurer BM 85 is applicable to arm circumferences ranging from 22 to 36 cm. The dimensions of the device are 180×100×40 mm, and the weight of the monitor without the cuff is 317 grams. The Beurer BM 85 is equipped with a rechargeable lithium-ion battery (3.7 V/400 mAh) that has a battery life of approximately 50 measurements.

Andersson Lifesense BDR 2.0 has a pressure range of 0 to 300 mmHg and a memory capacity of 60 measurements for 2 users. It can calculate an average value of the last 3 measurements. SBP and DBP as well as the heart rate are displayed on an LCD display. The monitor can identify an irregular heartbeat, which is then displayed with a symbol on the LCD screen. The included standard cuff for Andersson Lifesense BDR 2.0 is applicable to arm circumferences ranging from 22 to 32 cm. The dimensions of the device are 180×99×40 mm, and the weight of the device without the cuff is 300 grams. For the Andersson Lifesense BDR 2.0, 4 AAA-size alkaline batteries are needed. The device has an approximate capacity of 300 measurements.

### Statistical Analysis

We categorized the participants into low or high BP. Low BP was defined as SBP less than 140 mmHg and DBP less than 90 mmHg. High BP was defined as SBP 140 mmHg or above or DBP 90 mmHg or above. We used this classification as a BP of 140/90 mmHg is the diagnostic cut-off for the definition of hypertension in Europe [14]. Characteristics are presented as mean (SD) and n (%) for continuous and categorical variables, respectively. To assess whether there were any statistically significant differences between participants with low and high BP, Chi-square test was performed for categorical variables and 2-sided *t* tests were performed for continuous variables. Differences between the automatic monitors and manual BP measurements were calculated by subtracting the manual measurement from the automatic ones. Participants were divided into four categories classified by the differences according to whether they were within 5, 10, 15, or more than 15 mmHg. Separate variables were created for systolic and diastolic pressure. We conducted a sensitivity analysis to see if the result of Beurer BM 85 differed when including only the 155 participants in whom BP also was measured using Andersson Lifesense BDR 2.0. The Bland-Altman method was used to assess systematic differences in BP measurements between the manual and automatic monitors and as a graphical evaluation of the associations [15]. The difference in BP between the automatic monitor and the manual monitor was plotted on the y-axis and the mean of the two monitor measurements on the x-axis. The limits of agreement, equal to ±2SD of the mean difference, provide a measure of the variation. We assessed Spearman rank correlation coefficients between automatic and manual measurements to further examine the validity by measuring the degree of association. The significance level was set to .05. Analyses were performed using STATA 14 (Stata Corporation, College Station, TX, USA).

## Results

This study included 180 participants (119 men and 61 women) with a mean age of 60.1 (SD 11.4) years. Characteristics of all participants and according to low (n=83) and high BP (n=97) are shown in Table 1. Participants with low BP and high BP did not differ significantly with respect to age, gender, or smoking status. However, there was a statistically significant difference in body mass index (BMI; *P*=.02) between the high and low BP groups with higher BMI in the high BP group. In addition, there was a statistically significant difference in waist circumference (*P*=.04) for men with greater waist circumference in the high BP group. The mean BP values for all participants with the manual monitor were 138 (SD 15.5) mmHg for SBP and 83 (SD 9.7) mmHg for DBP. The Beurer BM 85 and Andersson Lifesense BDR 2.0 mean BP values are shown in Table 1.

The mean difference between the Beurer BM 85 and the manual BP monitor was 11.1 (SD 11.2) for SBP and 8.0 (SD 8.1) for DBP. The mean difference between the Andersson Lifesense BDR 2.0 and the manual BP monitor was 3.2 (SD 10.8) for SBP and 4.2 (SD 7.2) for DBP. The number of measurements that differed from the manual measurements by 5, 10, 15 or less, and more than 15 mmHg are shown in Table 2. For Beurer BM 85, 49.1% (83/169) of all measurements differed by 10 mmHg or less in SBP and 30.8% (52/169) by 5 mmHg or less for DBP. For Andersson Lifesense BDR 2.0, 69.7% (108/155) of all measurements differed by 10 mmHg or less in SBP and 49.0% (76/155) by 5 mmHg or less for DBP. In sensitivity analysis, the results of Beurer BM 85 did not differ when including only the 155 participants in whom BP was measured using both automatic BP monitors (data not shown).

**Table 1:** Characteristics of study participants by categories of low and high blood pressure.

Variable		Total (N=180)	Low blood pressure; <140/<90 mmHg (n=83)	High blood pressure; ≥140/or ≥90 mmHg (n=97)	*P* value^a^
Age (years), mean (SD)		60.1 (11.4)	59.2 (11.2)	60.8 (11.5)	.36
Male, n (%)		119 (66.1)	56 (47.1)	63 (52.9)	.72
Body mass index (kg/m^2^), mean (SD)		30.4 (5.4)	29.4 (5.1)	31.3 (5.4)	.02
**Waist circumference (cm), mean (SD)**					
	All	107.5 (14.8)	106.3 (13.7)	108.5 (15.6)	.34
	Male	110.1 (14.4)	107.3 (12.7)	112.7 (15.4)	.04
	Female	102.3 (14.2)	104.3 (15.8)	100.7 (12.8)	.33
**Smoking status, n (%) .54**					
	Never	72 (40.0)	35 (42.2)	37 (38.1)	
	Former	71 (39.4)	30 (36.1)	41 (42.3)	
	Current	20 (11.1)	11 (13.3)	9 (9.3)	
	Missing	17 (9.4)	7 (8.4)	10 (10.3)	
**Systolic blood pressure (mmHg), mean (SD)**					
	Manual	138 (15.5)	126 (8.7)	148 (13.0)	<.001
	Beurer BM 85^b^	149 (18.2)	137 (12.2)	159 (15.8)	<.001
	Andersson Lifesense BDR 2.0^c^	140 (18.5)	129 (11.0)	151 (17.5)	<.001
**Diastolic blood pressure (mmHg), mean (SD)**					
	Manual	83 (9.7)	78 (6.3)	88 (9.6)	<.001
	Beurer BM 85^b^	91 (10.1)	87 (7.8)	94 (10.6)	<.001
	Andersson Lifesense BDR 2.0^c^	87 (10.2)	82 (7.1)	91 (10.4)	<.001

^a^*P* value from Chi-square test or *t* test between groups of low blood pressure and high blood pressure.

^b^n=169.

^c^n=155.

**Table 2:** Validation results of the Beurer BM 85 and Andersson Lifesense BDR 2.0 (the number of measurements that differed from the manual blood pressure measurement by 5, 10, 15 or less, and more than 15 mmHg).

Variable		≤5 mmHg	≤10 mmHg	≤15 mmHg	>15 mmHg
**Beurer BM 85**					
	SBP^a^, n (%)	47 (27.8)	83 (49.1)	109 (64.5)	60 (35.5)
	DBP^b^, n (%)	52 (30.8)	105 (62.1)	146 (86.4)	23 (13.6)
**Andersson** **Lifesense** **BDR 2.0**					
	SBP, n (%)	68 (43.9)	108 (69.7)	129 (83.2)	26 (16.8)
	DBP, n (%)	76 (49.0)	123 (79.4)	147 (94.8)	8 (5.2)

^a^SBP: systolic blood pressure.

^b^DBP: diastolic blood pressure.

**Figure 1:**
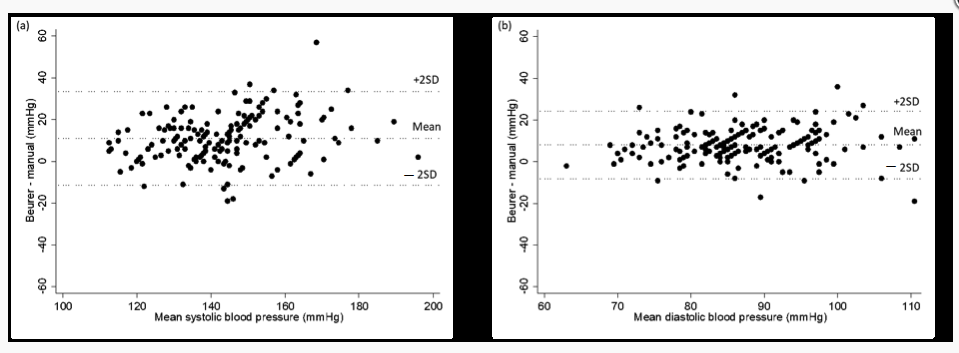
Bland-Altman plots of the differences between the Beurer BM 85 measurements and the manual measurements for systolic blood pressure (a) and diastolic blood pressure (b). The difference in blood pressure between Beurer BM 85 and the manual monitor is plotted on the y-axis and the mean of the two monitor measurements on the x-axis. Each data point represents one participant (n=169).

**Figure 2:**
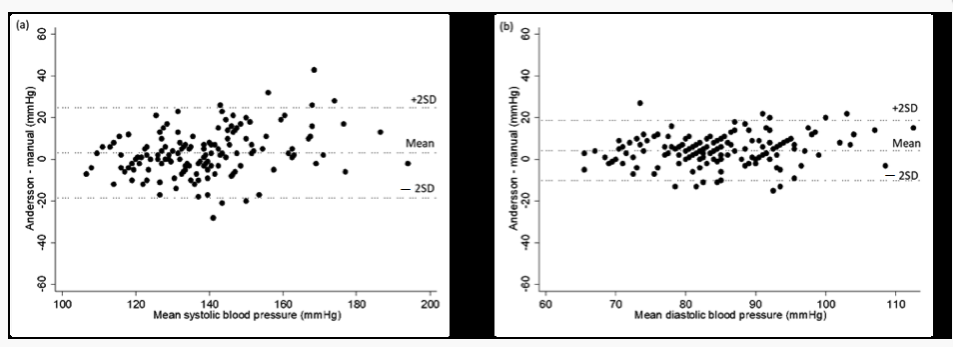
Bland-Altman plots of the differences between the Andersson Lifesense BDR 2.0 measurements and the manual measurements for systolic blood pressure (a) and diastolic blood pressure (b). The difference in blood pressure between the Andersson Lifesense BDR 2.0 and the manual monitor is plotted on the y-axis and the mean of the two monitor measurements on the x-axis. Each data point represents one participant (n=155).

The differences in SBP and DBP in relation to the mean between the automatic and the manual monitors are shown in Bland-Altman plots in Figures 1 and 2. Most data points fall within the limits of agreement (±2SD), although it should be noted that the limits of agreement were wide and individual differences are shown. The data points in all Bland-Altman plots show a consistent horizontal pattern around the mean of the y-axis, that is, no trend was identified, that is, the accuracy did not seem to be impacted by the level of SBP or DBP. All of the automatic BP measurements were significantly correlated with the manual measurements. The Spearman correlation coefficient was *r*=0.78 for SBP and *r*=0.71 for DBP for Andersson Lifesense BDR 2.0, and *r*=0.78 for SBP and *r*=0.69 for DBP for Beurer BM 85 (*P*<.001 for all).

## Discussion

### Principal Findings

The results of our study demonstrate that the mean difference between the manual and the automatic BP monitors Andersson Lifesense BDR 2.0 and Beurer BM 85 was small on a group level. The differences between the manual and automatic measurements were larger on an individual level.

There is a lack of studies validating automatic BP monitors in patients with type 2 diabetes. However, Masding et al [16] compared automatic home BP measurement and manual BP measurement with a previously validated 24-hour ambulatory BP monitor, which measured BP every 30 min during the day and every 60 min during the night, in 55 patients with type 2 diabetes. They found automatic home-measured BP superior to clinically measured BP. The mean difference between the automatic BP and the 24-hour ambulatory BP was 8.2 and 3.7 mmHg for SBP and DBP, respectively. The manual BP monitor compared with the 24-hour ambulatory BP monitor showed a mean difference of 10.9 and 3.8 mmHg for SBP and DBP, respectively. In our study, Andersson Lifesense BDR 2.0 showed a lower mean difference in SBP (3.2 mmHg).

Masding et al [16] have also predefined ranges for differences in BP that would be acceptable in clinical practice to be 10 mmHg for SBP and 5 mmHg for DBP. Comparing the results from our study, the Andersson Lifesense BDR 2.0 falls within these ranges more often than the Beurer BM 85. Although 69.7% (108/155) of automatic measurements performed using the Anderson Lifesense BDR 2.0 was within 10 mmHg from the manual measurement for SBP, only 49.1% (83/169) of automatic measurements with Beurer BM 85 were within this limit. For DBP, 49.0% (76/155) and 30.8% (52/169) were within 5 mmHg of difference for Andersson Lifesense BDR 2.0 and Beurer BM 85, respectively. On a group level, Andersson Lifesense BDR 2.0 meets the clinical ranges with a mean difference of 3.2 mmHg for SBP and 4.2 mmHg for DBP. Although their patient group was similar to ours, it should be noted that the studies differ in a number of aspects. First, in their study, BP was measured with the manual BP monitor at 3 different visits to the health clinic. Thereafter, the patients were instructed to use the automatic BP monitor at home at 3 specified times on 4 consecutive days, comparing it with the 24-hour ambulatory BP monitor [16]. In our study, BP was measured manually and by automatic monitors on 1 occasion only.

Several automatic BP monitors without a Bluetooth connection have been validated in the general population [17-21]. Takahashi et al [17] validated three different automatic BP monitors against manual BP monitoring. All three of them showed better accuracy than the two monitors we validated in this study. In our study, the percentage of measurements that differed from the control measurements by 5, 10, and 15 mmHg or less for the monitor with the best accuracy, Andersson Lifesense BDR 2.0, were 43.9% (68/155), 69.7% (108/155), and 83.2% (129/155) for SBP and 49.0% (76/155), 79.4% (123/155), and 94.8% (147/155) for DBP. For one of the monitors validated by Takahashi et al, the corresponding numbers were 72%, 92%, and 98% for SBP and 82%, 98%, 100% for DBP. However, they only included 33 participants from the general population and BP was measured with each of the monitors at 3 times on each participant, giving a total of 99 measurements [17]. These differences make it difficult to compare results across studies. Furthermore, in our study, the automatic BP measurements were significantly, with correlation coefficients of .69 or higher, correlated with the manual BP measurements. However, to the best of our knowledge, no correlation analysis has been conducted in any validation study of automatic BP monitors compared with manual BP measurement.

The large sample size is a notable strength of our study. Furthermore, the participants were recruited from 6 primary care centers located in different areas with diverse populations and levels of socioeconomic status. With a mean age of 60 years, the participants are younger than the general patients with type 2 diabetes in Sweden (68 years old) [22], which may be due to the inclusion criteria of having a smartphone. However, 8 of 10 Swedes have a smartphone [23]. Our study also includes a larger number of men compared with women. This may primarily be a reflection of the higher prevalence of diabetes type 2 among men compared with women in Sweden [24,25]. Nevertheless, this study includes more women than any previous studies validating automatic BP monitors [16,17].

There is a lack of automatic BP monitors validated in the large population of patients with type 2 diabetes who are likely to use automatic BP monitors in practice. Not only are patients with diabetes often familiar with digital devices that help them with better management, for example, devices for self-monitoring of blood glucose, they are also in need of controls for comorbidities, such as hypertension, on a regular basis. Today, the routine practice for screening of hypertension consists of multiple visits to the health care clinic for repeated BP measurement. Including home measurements in the decision making would not only be more cost-efficient but also provide more reliable measurements with white coat hypertension and masked hypertension in mind. The fact that BP measurements in our study were performed in a clinical setting and not in a home setting, and only once using each monitor, may be a limitation of our study. Though, as the aim was to validate the automatic monitors against the standard method for BP measurement, the manual and the automatic measurements were performed on the same occasion at a health care clinic.

Digital solutions could meet the needs and expectations of patients by making health care more accessible. Validated devices also ensure the quality of the care. However, if the BP monitors’ ability to transfer BP data via Bluetooth is to be used in future health care, it is important to emphasize that transfer of data to, for example, an electronic health record has to be compliant with security regulations and without the risk of privacy invasion of stored health data on the cloud. In addition, offering tech support, for example, an in-app chat-support, for various issues such as data transfer and uploading data could potentially increase consumer confidence in digital self-measurement devices. Gamification techniques such as visual feedback messages or a platform allowing communication could have potential to further increase the motivation of the patients to engage in their own health care [26]. For future patients, the BP data together with other variables could possibly allow for personalized lifestyle recommendations.

### Conclusions

In conclusion, no previous studies have validated automatic BP monitors with a Bluetooth connection in patients with type 2 diabetes. Our study shows that although the difference between the manual and the automatic BP monitors was greater on an individual level, the monitors are sufficiently accurate on a group level. Moreover, the Andersson Lifesense BDR 2.0 is more often falling within the BP ranges for what is acceptable in clinical practice compared with the Beurer BM 85.

## Abbreviations

BMI: body mass index
BP: blood pressure
DBP: diastolic blood pressure
LCD: liquid crystal digital
SBP: systolic blood pressure
